# A Method of Constructing Marine Oil Spill Scenarios from Flat Text Based on Semantic Analysis

**DOI:** 10.3390/ijerph17082659

**Published:** 2020-04-13

**Authors:** Kui Huang, Wen Nie, Nianxue Luo

**Affiliations:** 1 School of Geodesy and Geomatics, Wuhan University, Wuhan 420100, China; 2 State Key Laboratory of Information Engineering in Surveying, Mapping and Remote Sensing, Wuhan University, Wuhan 420100, China; niewen@whu.edu.cn

**Keywords:** scenario construction, marine oil spill, natural language processing, semantic clustering

## Abstract

Constructed emergency response scenarios provide a basis for decision makers to make management decisions, and the development of such scenarios considers earlier historical cases. Over the decades, the development of emergency response scenarios has mainly implemented the elements of historic cases to describe the grade and influence of an accident. This paper focuses on scenario construction and proposes a corresponding framework based on natural language processing (NLP) using text reports of marine oil spill accidents. For each accident, the original textual reports are first divided into sentence sets corresponding to the temporal evolution. Each sentence set is regarded as a textual description of a marine oil spill scenario. A method is proposed in this paper, based on parsing, named entity recognition (NER) and open information extraction (OpenIE) to process the relation triples that are extracted from the sentence sets. Finally, the relation triples are semantically clustered into different marine oil spill domains to construct scenarios. The research results are validated and indicate that the proposed scenario construction framework can be effectively used in practical applications.

## 1. Introduction

Oil pollution has been a major environmental concern since the 1920s [1] and a major menace to the environment because it severely damages the surrounding ecosystem [2]. Many oil spill pollution issues arise from the run-off of oil from onshore facilities and oil tanker spills during transportation [3]. Approximately 5.86 million tonnes of oil were lost as a result of tanker incidents from 1970 to 2019 [4]. The spilled oil is subjected to the process of weathering, in which the oil spreads and moves on the surface of the sea due to wind and currents. During this movement, the oil can undergo chemical and physical changes [5]. Due to the sophisticated hydrogeological and biological factors that influence oil movement, oil pollution is often characterized as an unconventional emergency and is associated with many new and unidentified risk factors, such as those related to spill magnitude uncertainties, which may not be foreseeable in the disaster preparedness stage [6]. Therefore, scenario analysis is of interest because it addresses variable uncertainties. Scenario analyses of the characteristics of historical data have been successfully applied in many fields, including in the long-term forecasting of nonresidential gas consumption [7], the estimation of the potential for reduction in CO_2_ emissions in China’s iron and steel industry [8], the simulation of water scarcity in the Beijing-Tianjin-Hebei (BTH) urban agglomeration [9], and the modeling of some economic services [10].

One major issue in early scenario research concerned scenario building. Early studies of scenario building used the Delphi-based method, which aims to increase the creativity, credibility, and objectivity of scenarios [11], and the cross-impact analysis method [12], which provides an orderly method of structuring to help determine how relationships between events impact resulting events and to reduce unwanted events in the future. Recently, the Strategic Sciences Working Group (SSWG) assessment involved the parameters of the Deepwater Horizon oil spill; specifically, parameter subsets were used to develop a detailed “chain of consequences” and assign a level of uncertainty to each element in each chain [13]. These methods are effective in scenario construction for specific events, but decision makers are faced with the challenge of mitigating not only single hazards and the related risks but also chains of events considering systemic interrelations [14]. By using a series of possible marine oil spill scenarios, system operators and participants can investigate the influence of oil spills on cost and reliability to improve decision making in an uncertain environment. There is still uncertainty in this approach, but a practical method for pre-constructing many marine oil spill scenarios does not exist. This paper discusses, from a practical point of view, the modeling needs and issues to be considered in the development of scenario construction tools for preprocessing historical marine oil spill case data to support decision makers in their preparedness and response strategies. A key aspect of scenario construction is describing the scenarios and scenario relationships for a given event considering the real-time status of the event and existing historical case data.

There are several formats used to store historical case data for marine oil spills, such as textural narratives, storyboards, video mock-ups, and written porotypes. As a matter of course, a textural narrative, such as a news report, has advantages in both authenticity and quantity, and such narratives are easy to obtain. Recent developments in text-based methods have heightened the need for natural language processing (NLP). NLP techniques have been applied in various areas, including machine translation, email spam detection, information extraction (IE), summarization, and question answering. Some applications include extracting knowledge and generating object-oriented models from text [15,16,17], producing games with dialogue management [18], and generating games from flat stories [19]. Thus, it is feasible to perform marine oil spill scenario construction based on NLP techniques. In this paper, NLP is employed to extract the necessary variable information and event relationships from text to build a marine oil spill scenario, mainly on the basis of five influencing components: space, time, hazard, exposure, and human behavior [14]. Our main contribution is the proposal of a practical framework based mainly on NLP tools and semantic clustering for constructing marine oil spill emergency scenarios from flat text reports.

The remainder of this paper is organized as follows. Section 2 presents a brief introduction to the fundamental theory of the proposed framework. Section 3 shows the experimental results to verify the effectiveness of the proposed method. Finally, a brief discussion is given, and the study conclusions and proposed future work are discussed.

## 2. Materials and Methods

**Figure 1:**
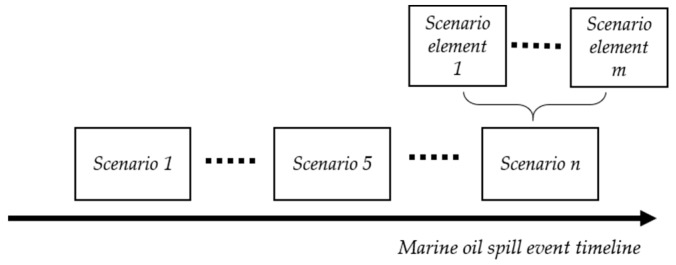
Hierarchical event–scenario instance–scenario element instance relationships**.**

The scenario concept was first proposed in 1967, and there are two main consistent conceptions of scenarios—(1) a scenario is a rational description of the future evolution of an event and (2) a scenario is not based on pure speculation or prediction but a projection of an event with credible, possible, and relevant features [6]. This paper considers a typical scenario is a collection of numerous similar scenario instances, as well as seeks to extract information related to the marine oil spill domain from text. From the perspective of scenario instance collection, a single scenario instance can also be considered as an untypical scenario. For an entire event, following the “event–scenario instance–scenario element instance” structure shown in Figure 1, event profile control can be divided into multiple scenario instances, and each scenario instance is a combination of multiple scenario element instances. This kind of structure divides a unique event into many relatively independent events with shared element instances.

**Figure 2:**
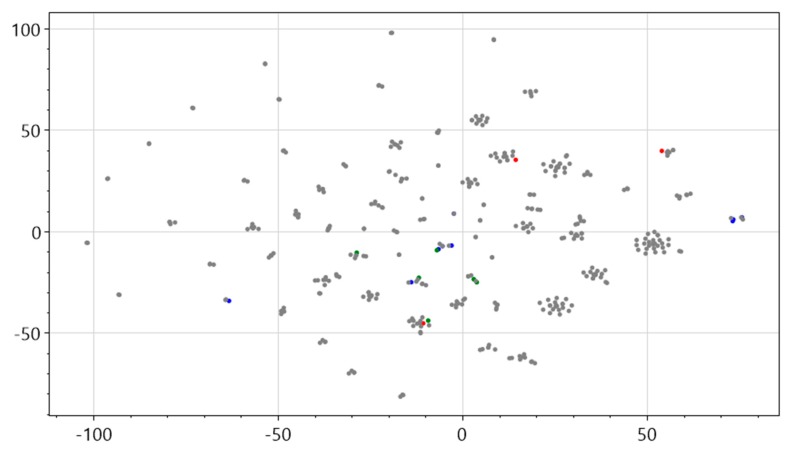
Visualized word vector result**.**

A major advantage of this structure is that it can record scenario information and preserve the temporal relationships between scenarios. The first step in this process was to analyze the relationships among subevents throughout the temporal evolution (timeline) of a marine oil spill event. In an attempt to emphasize each subevent, the sentences related to a subevent were aggregated to form a sentence set, which is a pure text form of a scenario. Each sentence set was then analyzed to extract relation triples using the NLP method. The final stage of the process involved semantic clustering within a pre-established marine oil spill domain to find the domain-related scenario element instances. The premise of this framework is that high-dimensional word embedding clustering can be used to classify the words from the text reports into appropriate scenario elements for marine oil spill accidents. Prior to presenting the framework, some expert knowledge is summarized to construct typical scenario elements of marine oil spill events. An experiment is performed to observe the spatial clustering relations between the word vectors related to the scenario elements and the word vectors extracted from the short text report of the historic case BRAER. The result is visualized using t-distributed stochastic neighbor embedding (t-SNE) [20] for dimensionality reduction in Figure 2.

In Figure 2, the red dots represent the crude oil scenario element with three words “oil,” “fire,” and “explode,” corresponding to the hazard; the blue dots represent the marine organism scenario element, coastline and marsh with words “fish,” “lobster,” “seabird,” “algae,” “marsh,” and “coastline,” corresponding to exposure; and the green dots represent the rescue scenario element with words “rescue,” “firefighter,” “recycle,” “recover,” “rinse,” and “clear,” corresponding to human behavior. The word vectors show obvious clustering characteristics after dimensionality reduction via t-SNE. In this figure, words in the same cluster have strong semantic relations. Therefore, this research assumes that the high-dimensional words extracted from the text reports can be classified into the appropriate scenario element domains in relation to oil spill events by clustering with the word associated with the pre-established scenario elements.

**Figure 3:**
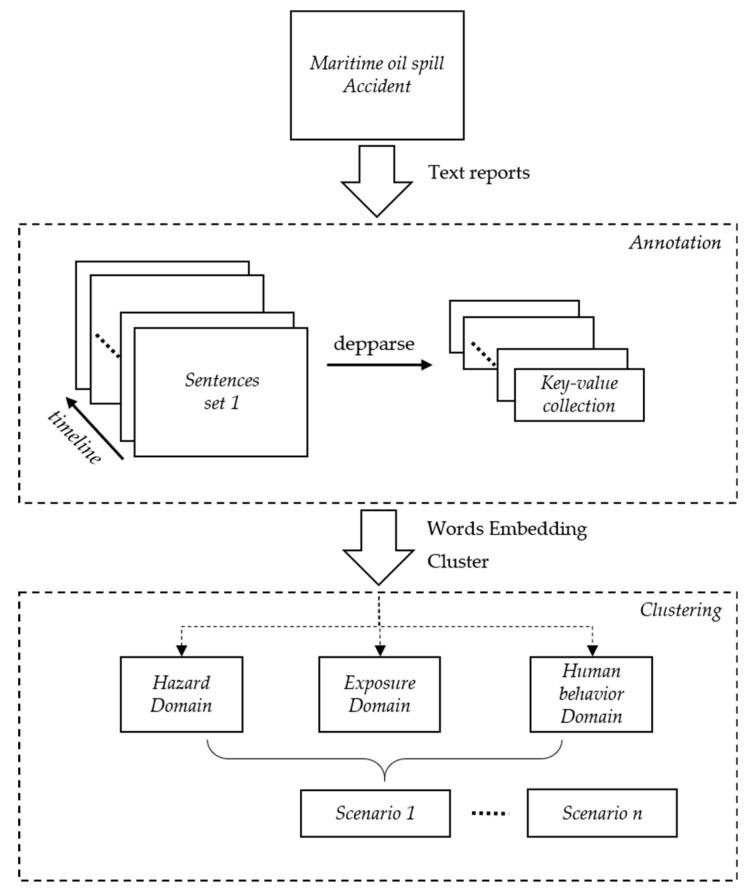
Overview of the scenario construction framework**.**

The following scenario construction framework based on this hypothesis is shown in Figure 3. A text report is first processed into sentence sets according to the timeline of the marine oil spill event. Each sentence set represents the situation in which the event evolves. Then, the sentence set is processed to obtain a relation triple structure in the form of “relation (entity, entity/value).” Finally, the relation triples are processed with the pre-established marine oil spill domains based on semantic clustering to generate object-oriented structure instances. The marine oil spill scenario consists of five components—space, time, hazards, exposure, and human behavior effects. Space and time coincide with the minimum space and time units of an event. Hazards refer to the triggering factors that lead to disasters, which may cause property losses, casualties, the destruction of resources and the environment, chaos in social systems, and other effects. Exposure refers to the main portion of human society directly affected and damaged by disasters, including groups and individual human beings, as well as economic, environmental, and social factors related to human beings. Human behavior effects, such as rescue activities, can significantly influence the evolution of events [21].

### 2.1. Scenario Element Domain

This section provides detailed illustrations of the pre-established marine oil spill domain. Each marine oil spill scenario is composed of some instances of the five components. The hazard component is the time-space distribution of the intensity of a given event with an assigned occurrence probability at a given time and in a given geographical area. The exposure component is the distribution of the probability that a given element (including people, buildings, infrastructures, the economy, or the environment) is affected by a disaster. Human behavior can strongly affect the final result of a disaster and is related to the effective implementation of preparedness actions, such as evacuation and rescue procedures. Table 1 shows the types of scenario element instances defined in this paper, which are used to represent different domains of marine oil spills.

**Figure 4:**
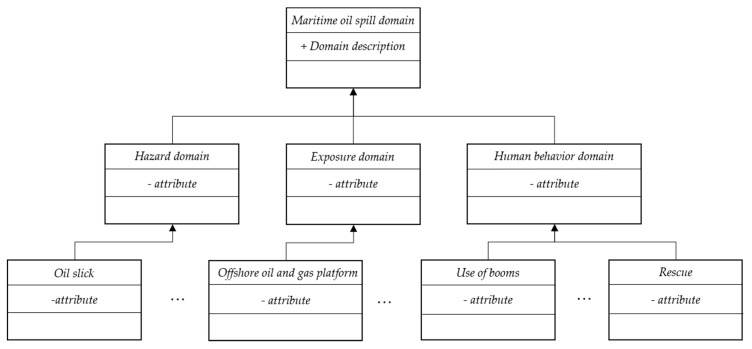
Object-oriented (OO)-based structure of the marine oil spill domain**.**

The goal of this research is to construct emergency response scenarios to support decision-makers in their preparedness and response, while exposure and human behavior in the scenario are the key points to assist in decision making. The exposure in this study includes some aspects that may be directly affected by oil spills, such as gas platforms and marine organisms (such as fish, birds, lobster and algae), and some facilities that indirectly surround coastal cities, such as fisheries and mariculture, coastal city roads, coastal city power supply systems, and subsea tunnels. Human behavior may change the subsequent scenario evolution, such as the behavior of dealing with spilled oil and firefighting. In this research, each domain is the abstraction of a scenario element instance. One of the most well-known methods for modeling this abstract structure is the object-oriented (OO) method, the structure of which is shown in Figure 4. 

The attribute “Domain description” in the marine oil spill domain is used to describe this domain and distinguish it from those of other hazards, as formally described by some professional vocabulary. The components in the marine oil spill domain consist of hazard, exposure, and human behavior, which are also formally described by professional vocabulary and just differ in scope. The scenario element instance domain defines detailed attributes; for instance, the “oil slick” domain has an attribute named “color.”

### 2.2. Relation Triple Extraction Based on Stanford NLP Tools

The marine relation triples are the smallest units that can express the relationship between a target object and its value. The original relation triples are extracted based on universal dependencies (UDs) and enhanced++ UDs, which are used to analyze the syntactic sentence structure. In this paper, the relation triple has a fixed structure of “relation (entity, entity/value)” based on a comprehensive analysis of NER, dependency (UD, enhance++ UD), and open information extraction (OpenIE).

In this paper, the Stanford CoreNLP toolkits are employed to annotate the text [22]. Stanford CoreNLP is a Java (or at least JVM-based) annotation pipeline framework that provides most of the common core NLP steps, from tokenization to coreference resolution. Our proposed method uses the annotators part-of-speech (POS) tagger [23,24], NER [25], parser [26], and relation extractor [27] tools.

#### 2.2.1. Event Splitting

A marine oil spill event can be regarded as a series of typical element-based scenarios related by a single timeline of events. The goal of event splitting is to extract a single chain of events with the NLP annotator tool from text reports according to the time evolution of the event. The concept of NLP focuses on understanding inputs in the form of natural language and producing an interpretation of this language using a computer. In the scope of this work, a scenario is a specific sequence of events that can be run in a simulation and is constructed to observe causal relations.

SUTIME [28] is part of named entity recognition (NER) and is employed to recognize the time entity of a text report. The text always has a narrative-like stylistic form, and the main content involves people, narration, scenes, objects, the experiences of people, and the development of and changes in different things. Every marine oil spill report has a main internal timeline. The goal is to divide the sentences in a report according to the evolution time points of the event and reorganize them into sentence sets. To eliminate description interference and generate specific time points, the rules are established as follows:

1. All timestamps must be in the range of the event start time and end time. Some timestamps in the report are not pivotal points in the evolution of an event. One such example is “They claim that a large military exercise will be held here in 20 days.” In this sentence, the reference to “20 days” will be recognized as “TIME” and normalized to “2019-12-21” if the real marine oil spill event started on 1 December 2019. If this timestamp is beyond the end of the oil spill event, it is evident that this is not a critical timestamp for this oil spill event; as a result, it can be neglected. This rule is used to ensure that all timestamps extracted from a report are valid event evolution timestamps.
2. Partially specified times and relative times need to be converted to absolute times. SUTIME recognizes both relative times, such as “next Monday,” and absolute times, such as “12 January 1999.” SUTIME also handles partially specified times, such as “the nineties.” The relative and partially times specified expressions cannot be directly used in historical comparisons; therefore, they must be converted to absolute times, such as converting “Saturday morning” to “2011-09-24 6:00.” The original normalized expression of this result is “2011-09-24TMO.”
3. The relationship between two scenarios is that the earlier-occurring scenario points to the latter scenario.

#### 2.2.2. Relation Triple Extraction

**Figure 5:**
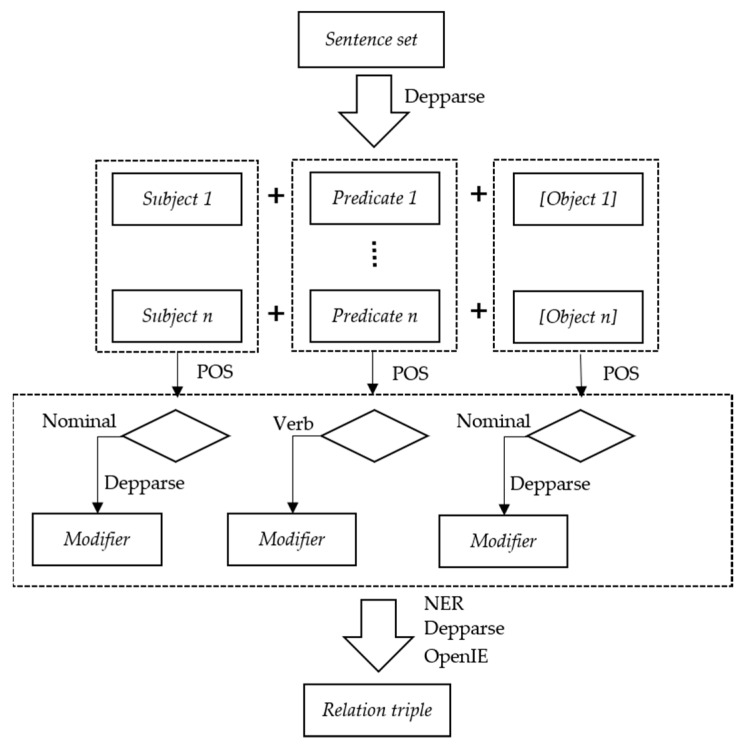
Overview of the relation triple extraction process

An overview of the relation triple extraction process is shown in Figure 5. NER labels sequences of words in a text, which are the names of things, such as the names of people and companies, genes and proteins, or numerical entities (money, numbers, dates, time, durations, and sets) [25]. This step focuses on three classes (persons, organizations, and locations) of recognition. The parser step aims to determine the grammatical structure of sentences, namely, determining which groups of words go together and which words are the subject or object of a verb. The parser annotates original sentences with UDs to provide simple descriptions of the grammatical relationships in a sentence that can easily be understood and effectively used by people without linguistic expertise who want to extract textual relations. The OpenIE annotator extracts open-domain relation triples representing a subject, a relation, and an object associated with the relation. Even when no specific domain and no training data are available, this method can easily extract the information required from open-domain relation triples. At the beginning of the process, the sentence set is processed by a syntactic dependency parser to generate dependency annotations, which makes it easy for developers to recognize the subject (S), predicate (P), and object (O) in a sentence. Two sentences are selected from marine oil reports to explain the dependency annotation results, as shown in Table 2. After collection, the subject, predicate, and object are further extracted.

If the subject is nominal, that is, the subject POS tag type annotated by the POS tagger annotator is a singular or mass noun (NN), plural noun (NNS), singular proper noun (NNP), proper plural noun (NNPS), or personal pronoun (PRP), further extraction will occur. For example, in sentence 1 of Table 2, the POS tag of the subject “ships” is NNS, which satisfies the nominal subject condition. Assuming that the “ships” are a critical element of the marine oil spill scenario, the next step is to find all related modifiers. In this case, the modifier dependency type is a numeric modifier (nummod) without units, and the enhance ++ UD relation triple is “nummod (ships, 2).” Table 3 shows the syntactic modifier relation used in this paper.

Different from the subject, the POS of the predicate is generally a verb, which is used to describe the subject’s action or individual state. The POS tag of the predicate is always a verb base (VB), verb past tense (VBD), verb gerund or present participle (VBG), past verb participle (VBN), verb non-third-person singular present (VBP), or third-person singular present verb (VBZ). In addition, the expletive (expl) relation needs to be excluded because expletives are nominals that appear in an argument position in the predicate but do not themselves satisfy any of the semantic roles of the predicate. For example, in the sentence “There is a ghost in the room,” “There” and “is” have expletive relations, and “is” is an actor in the predicate in this sentence; however, “is” here does not represent an action or state.

The object, also known as the addressee, refers to the receiver of an action (predicate). For example, in sentence 2 of Table 2, the subject ‘authority’ and object ‘statement’ are related by “released.” In addition, the objects also have modifiers, and the corresponding relations are the same as the syntactic modifier relations in Table 3. In the next phase of the process, these syntactic modifier relations are processed to generate relation triples through further analysis. Moreover, OpenIE participates in extracting the relation triples as supplementary information. The OpenIE annotator extracts open-domain relation triples representing a subject, a relation, and the object of the relation. In addition to extracting open-domain relation triples, the annotator produces several sentence fragments corresponding to entailed fragments from the given original sentences. For example, in example sentence 2 of Table 2, the extracted relation triple is “released (Kamarajar port authority, press statement).” The “Kamarajar port authority” and “press statement” are recognized as entities, and the collection of extracted relation triples represents the relations among entities. The relation triples extracted from the two example sentences are shown in Table 4.

### 2.3. Semantic Clustering

In Section 2.1, the definition of an oil spill scenario can be described by five components: time, space, hazard, exposure, and human behavior. To construct this form of scenario, this research has to eliminate the irrelevant relation triples extracted from the text reports by analyzing the correlations between words and marine oil spill events, especially by comparing the similarity between the relation triples and the marine oil spill domains. The main steps are as follows:

1. Clustering the extracted relation triples and marine oil domains; semantic clustering is employed to explore the entities involved in relation triples that are semantically closest to the marine oil spill domain.
2. Semantic matching of the attributes in the marine oil spill domain with the relations of the extracted relation triples. The attributes in each pre-established domain are fixed names with specific meanings, such as “color” and “weight.” Additionally, semantic matching aims to find the attributes in the marine oil spill domain with semantic similarity.

Global Vectors for Word Representation (GloVe) is an unsupervised learning algorithm for obtaining vector representations of words. Training is performed based on aggregate global word–word co-occurrence statistics from a corpus, and the resulting representations reflect the interesting linear substructures of the word vector space [29]. Thus, the model produces a word vector space with a meaningful substructure.

The main purpose of the GloVe model is to establish the matrix of word-word co-occurrence counts denoted by X, with entries $X_{ij}$ that tabulate the number of times word j occurs in the context of word i. The GloVe model is more efficient than Word2Vec because it is trained only on global nonzero matrix elements $X_{ij}$. The model uses the least squares method as the loss function and adds offsets to the rows and columns in the co-occurrence matrix X. Specifically, the loss function is

(1) $$L=\overset{\left|V\right|}{\underset{i,j=1}{\sum }}f\left(X_{ij}\right){\left(w_{i}^{T}w_{j}+b_{i}+b_{j}-logX_{ij}\right)}^{2}$$

where |V| is the size of the dictionary, $w_{i}$ is the word vector of the target word, $w_{j}$ is the word vector of the contextual word, $b_{i}$ and $b_{j}$ are the offset values of the Xth row and column of the co-occurrence matrix, and f(x) is a weighting function used to attenuate the low-frequency word pairs counted from the corpus to reduce the error caused by low-frequency noise, which is defined as

(2) $$f\left(x\right)=\{\begin{array}{c} {\left(\frac{x}{x_{max}}\right)}^{\alpha },\;if\;x\leq \;x_{max}\\ 1,\;otherwise \end{array}$$

where $x_{max}$ and α are assigned values of 100 and 3/4, respectively, in all the experiments [29]. The Stanford NLP group also provides pretrained GloVe models. In this paper, the following pretrained model: Wikipedia 2014+ Giaword5 (6B tokens, 400 vocab, uncased, 50d vectors) is used to convert words to vectors. For example, the word “oil” is converted into a vector as (0.194, 0.993, 0.003, …). In embedding spaces, semantically similar words are likely to be clustered together and form semantic cliques, which reflects the ability of word vectors to extract information related to oil spill scenarios from raw text. The scenario information can be extracted from the text based on word clustering by advance definition words, which consist of hazard, exposure, and human behavior domain.

Each marine oil spill domain has a domain description, and the domain description consists of several keywords. These domain description words are included in clustering to find the most relevant domain for the semantics of the relation triple. Data clustering is one of the most important and popular data analysis techniques for understanding data. It refers to the process of grouping objects into meaningful subclasses (clusters) such that members of different clusters differ as much as possible. The density-based clustering approach is one of the most popular paradigms, and the most famous algorithm of this kind is DBSCAN [30]. However, DBSCAN has disadvantages for high-dimensional data due to the so-called “curse of dimensionality,” whereas the NQ-DBSCAN variant can handle this problem well [31]. On the basis of NQ-DBSCAN, an improved method of finding the most semantically similar scenario element domains to the words extracted from text reports is proposed. The unclassified points constitute a collection of relation triple entity words W, which are extracted from text reports, and a collection of scenario element domain words E, which have been pre-established as part of the domain description step. Before clustering, the scenario element domain words are marked according to their domain types. When performing NQ-DBSCAN, a word $w_{i}$ is selected randomly from the set of relation triple entity words W. If $w_{i}$ is a core object, then it satisfies

(3) $$\left|N_{\epsilon }\left(w_{i}\right)\right|\geq MinPts$$

The algorithm needs to create two arrays, one to store $w_{ki}\in W$ and one to store $E_{ki}\in E$. Here, MinPts is the minimum number of points required to form a cluster. When cluster C {c| c$\in w_{k}\;\cup \;$ c$\in e_{k}$} is finished being expanded, the next step is simply need to recalculate $N_{\epsilon }\left(w_{ki}\right)\leq \epsilon$ from the reference points$\;e_{k}$, where $N_{\epsilon }\left(w_{ki}\right)\leq \epsilon$ means that the word collection satisfies the following condition:

(4) $$\left\{o|o\in W\cup E,distance_{w_{i},o}\leq \epsilon \right\}$$

The word$\;w_{ki}$ belongs to the domain that has the most tokens in $N_{\epsilon }\left(w_{ki}\right)$. The main steps are shown in Algorithm 1.
**Algorithm 1:** Improved NQ-DBSCAN. Input:W: relation triple entity word vectors E: domain word vectors ϵ: maximum distance MinPts: minimum number of points to form a cluster Output: w_i_ and the domain of each word Tw_i_
Initialize cluster id C=0 for each unclassified word Tw_i_ do // according to [31] $N_{\epsilon }\left(w_{i}\right)$=RangeQuery ($w_{i},2\epsilon$) if (|$N_{\epsilon }\left(w_{i}\right)|\geq \mathrm{MinPts}$ then  dists← all distances from $w_{i}$ to $N_{\epsilon }\left(w_{i}\right)$
 |distArr, pLoc|=sort(dists)  if distArr[MinPts]≤ϵ then   // mainly according to [31], return two arrays    drPts,$w_{k}$,$e_{k}$= ImprovedExpandCluster ($w_{i}$, pLoc, distArr, ϵ, MinPts)    C←C+1  else    set {o| o$\in {\mathrm{pLoc}\;\mathrm{and}\;d}_{w_{i},o}<\mathrm{distArr}\left(\mathrm{MinPts}\right)-\epsilon$} as noise  end if else  set {o| o$\in N_{\epsilon }\left(w_{i}\right)$} as noise end if //search for the most semantically similar domain  for each word $w_{\mathrm{ki}}$ do  $N_{\epsilon }\left(w_{\mathrm{ki}}\right)=\mathrm{RangeQuery}\left(w_{\mathrm{ki}},\epsilon \right)$ by given points $e_{k}$
 ${\mathrm{Tw}}_{\mathrm{ki}}$ is the maximum number of marked domain words in $N_{\epsilon }\left(w_{\mathrm{ki}}\right)$
 end for end for

Each marine oil spill domain is represented by a particular OO-based structure, which consists of attributes. The relation triple information is used to calculate the semantic similarity with the average of the word vectors of each attribute in the domain. A single-attribute word is denoted by $w_{i}=\left(x_{1},\;x_{2},\ldots ,x_{n}\right)$, where $w_{i}$ is also an n-dimensional vector of a “relation” word from a relation triple. The similarity between $w_{i}$ and $w_{j}$ can be measured as follows:

(5) $$s\left(w_{i},w_{j}\right)=\frac{w_{i}\cdot w_{j}}{|w_{i}|\left|w_{j}\right|}$$

According to our experience, when $s\left(w_{i},w_{j}\right)$ is higher than 0.75, the relation triple has a strong correlation to the attribute of the domain.

## 3. Experiments and Results

The proposed methodology is intended to extract information from a variety of marine oil spill-related documents. In this phase, the authors tested the proposed method on real historical case data. An evaluation of scenario element instances is conducted by comparing the extracted information with a “standard.” The “standard” includes all instances of the target information in the regulatory text source and is manually compiled by domain experts. The recall is defined as the ratio of $I_{c}$ to $I_{t}$, where $I_{c}$ is the correct sum of the valid attributes of the extracted scenario element instances and $I_{t}$ is the total number of attributes of the scenario element instances for the event extracted manually.

(6) $$P=\frac{\;I_{c}}{I_{t}}$$

The method can be evaluated in terms of precision, and satisfactory performance supports the validity of the proposed approach and methodology.

### 3.1. Selection of Marine Oil Spill Historical Cases

The International Tanker Owners Pollution Federation Limited (ITOPF) is a not-for-profit organization that was established on behalf of the word’s shipowners to promote an effective response to marine spills of oil, chemicals, and other hazardous substances. The International Oil Pollution Compensation Funds (IPOC Funds) is a program that provides financial compensation for oil pollution damage that occurs in the member states resulting from spills of oil from tankers. Since its establishment, the IOPC funds program has been involved in 150 incidents of varying sizes worldwide. To apply the proposed method for marine oil spill scenario construction, ten major spills were chosen from among the ITOPF historical cases recorded since 1967. The text reports for these ten major spills were mainly collected from the ITOPF and IOPC Funds, which incorporate Wikipedia content; the events with external Wikipedia links are listed in Table 5.

### 3.2. Experimental Results

The event splitting experimental results are shown in Table 6. The accuracy of the “event scenario” process is satisfactory, and only the “HAVEN” case was associated with an error. Through the analysis of the original text report, the cause of the error was related to the treatment of “on day two” by SUTIME. Notably, the phrase “on day two” cannot be successfully recognized as a normalized time expression.

In the following experiment, the phrase “on day two” replaced with “on the next day,” and errors associated with the number of scenarios are eliminated. The correct number of element instances in each scenario is determined according to three components: hazards, exposure, and the human behavior.

**Figure 6:**
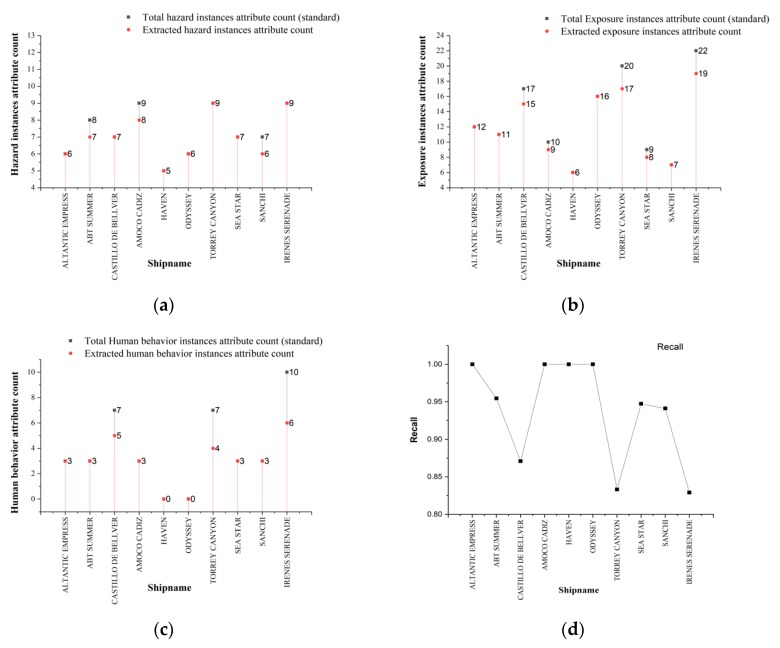
(**a**–**c**) show the statistical results for the attribute counts of the three components and (**d**) shows the recall of the proposed framework for each marine oil spill event.

In practice, a text report does not always repeat written scenario element instances in the sentence sets of subsequent scenarios, but scenario element instances should appear in subsequent scenarios based on reasoning. For example, the initial scenario sentence set describes the state of the ship, but the subsequent scenario sentence set focuses on depicting personnel rescues and does not include a text description of the ship, which may lead to missing the “transport vessel” element instance in this subsequent scenario. From the perspective of the entire event, the following scenario should, by default, include the exposure scenario element instance “ship.” To avoid this error, a warehouse is prebuilt for each oil spill event; the warehouse retains the scenario element instances from the previous sentence set for a given marine oil spill event according to the time evolution of the event. When processing a subsequent scenario, the scenario element instances in the warehouse are added to the current scenario by default. After the subsequent scenario is processed, the warehouse needs to be updated according to the scenario element instances extracted from the previous scenario. The subsequent scenario can inherit scenario element instances in the warehouse similar to member variables inheritance in OO. Therefore, when calculating the accuracy of the method, we count the scenario element instances from the perspective of the entire event instead of repeatedly counting the scenario element instances in each splitting scenario. The statistical results are as follows:

In the figure above, the number on the red line in Figure 6a–c is the statistical result for each event, where the attributes of the extracted scenario element instances that meet the conditions of the hazard, exposure, and human behavior domains after semantic clustering and semantic matching are retained. Moreover, the number on the black line is the statistical result for each event determined manually for the three components. Notably, Figure 6a shows that when the total number of attributes is less than seven, the proposed method can achieve satisfactory accuracy because the pre-established domain defines only two types of scenario elements for hazards and both have clear semantic distinctions from those in other domains.

Compared to the results shown in Figure 6a, the results in Figure 6b display more errors. Based on an analysis of the original text reports, the main reason for these errors is that some implicit meanings in the text cannot be directly extracted by parsing and OpenIE, and the appropriate relation triples cannot be generated. For example, an error occurs in the case “AMOCO CADIZ,” mainly because the phrase “fishermen in the area caught fish with skin ulcerations and tumors” does not imply that “fishermen” are also victimized, and this triple cannot be directly extracted from dependencies and OpenIE. The description of the human behavior domain is relatively fuzzy. For the human behavior component, the relation triples that can be extracted are generally related to the keyword “rescue.”

Figure 6d shows the overall recall of the proposed method for each marine spill event. No significant evidence indicates that the recall is correlated with the spill size in the proposed method. In summary, these results suggest that domain description words with effective discrimination ability can make semantic clustering and semantic matching more accurate.

## 4. Discussion

The emergency response scenario is not a typical case, nor is it a projection of specific events, the core of which is how to prepare before “assuming the accident has happened.” The main purposes of constructing a marine oil spill emergency response scenario is to assist decision-makers in making decisions, implying more focus place on the menace to the ecosystem caused by human behavior. Ecosystems deliver and sustain a wide range of benefits that contribute to people’s well-being and livelihoods, which can be valued in economic terms [32]. In this case, the appropriate scenario elements are used to measure the oil spill pollution from the historical oil spill scenario to help decision-makers analyze the consequences of human behavior.

**Figure 7:**
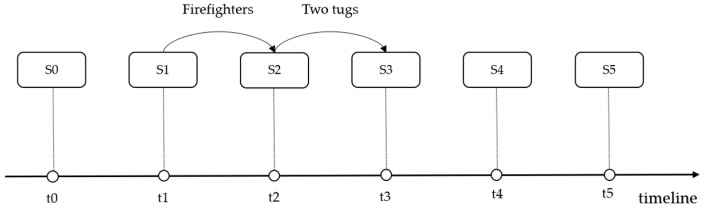
The scenario instances of the ATLANTIC EMPRESS.

For example, the proposed method extracted six scenarios from the case “ATLANTIC EMPRESS,” and the chain structure is shown in Figure 7. At the initial scenario S0, the ATLANTIC EMPRESS collided with the Aegean Captain. With the evolution of the event, scenario S0 transitioned to S1, and large fires that began on each ship were soon beyond the control of the crews, who then abandoned their ships. In scenario S1, the firefighters (rescue team) were involved in the event, and their involvement illustrated decision-making behavior that may change the subsequent scenario evolution. With the fire under control, scenario S1 transitioned to S2, and two tugs (rescue team) participated (rescue team) by towing the burning ship ATLANTIC EMPRESS farther out to sea. A week later, the ATLANTIC EMPRESS was still burning. With the occurrence of an explosion, scenario S2 transitioned to S3. The next day, with an even larger explosion that occurred (scenario S4), the rate of the oil spill increased to 7000~15,000 gallons per hour. Finally, the ATLANTIC EMPRESS sank (scenario S5). The oil spill emergency response scenario can be measured by an evaluation model, such as the DLSA, with an index that consists of the spilled oil amount, integrated sensitivity, weather, ship type, toxicity, flammability, persistence and thread grade [33]. According to the DLSA model, the risk of oil spill pollution was reduced when the scenario evolved from scenario S1 to scenario S2. However, the risk dramatically increased when scenario S2 transitioned to scenario S3. Consequently, both of the rescue actions in this case should be part of the decision. Therefore, the accumulation and improvement of these decision-making behaviors and their related scenarios can assist decision makers in giving more adequate consideration to similar situations that might arise.

**Figure 8:**
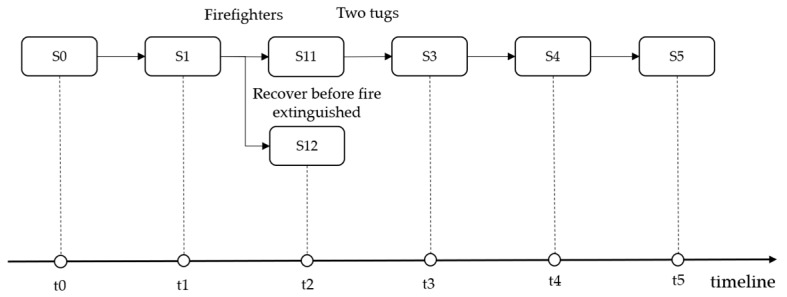
The Expansion of the ATLANTIC EMPRESS scenario instances chain.

Although an oil spill can occur in different parts of the world, oil spill events share similarities regarding the hazard, exposure or human behavior elements. Another case concerns the “SEA STAR,” which was a supertanker that spilled some 115,000 tons of crude oil into the Gulf of Oman in 1972. In the reports, the SEA STAR caught fire after colliding with the Brazilian tanker Horta Barbosa. However, the repair of the SEA STAR failed without extinguishing the fire. Eventually, with serval explosions, the vessel sank in the Gulf of Oman. From the perspective of exposure, the SEA STAR and ATLANTIC EMPRESS were oil tankers that both caught fire after the collision. Moreover, the scenario of the SEA STAR resembles such as scenario S1 of the ATLANTIC EMPRESS in that there were no firefighters to extinguish the fire successfully. In this case, the scenario chain of the ATLANTIC EMPRESS can be extended by connecting the scenario of the SEA STAR to S1, as shown in Figure 8. Consequently, as the historical scenarios increase, the branches of the tree become richer, which may assist decision makers in preparing for a marine spill event.

Furthermore, there are also recent studies on the automatic extraction of relation pairs as well as Relata, a database of natural language relation pairs. In our research, we have adopted an expert-system-based scenario model and combined it with standard off-the-shelf NLP tools to construct a specific scenario of an event with environmental impact. Our research has focused on the application of high-dimensional density clustering to the elements of marine oil spill scenarios. In our future work, we will further explore ways to improve the proposed method and reduce errors, thereby improving the extraction results, such as applying an ontology for the definition of specific domains and working under the framework of the DARPA LORELEI program to quickly extract critical information [34].

## 5. Conclusions

This paper has presented a framework that is mainly based on NLP and semantic clustering for extracting scenario element instances from marine oil spill event reports to construct emergency response scenarios for decision making. The paper also demonstrated a process of constructing scenario chains from historical cases. With the increase in the scenario chain, some of the chain can be expanded to a scenario tree by analysis of the hazard, exposure or human behavior. These branches occur mostly because of human behaviors, providing significant and intuitive help for decision making. However, the process of scenario chain expansion to a scenario tree depends mainly on manual work. Therefore, an effective method is urgently needed to accommodate a large number of scenarios, which is the key point we will study in the future.

## Figures and Tables

**Table 1:** Summary of scenario elements and domains related to marine oil spill events.

Scenario Element Instance Type	Domain
Hazard	Oil slick Crude oil
Exposure	Offshore oil and gas platform Marine organisms Fisheries and mariculture Costal city power supply system Coastal city road Subsea tunnel Port infrastructure Oil tanker
Human behavior	Use of booms Use of dispersants Use of skimmers Use of sorbent materials Clean-up of oil from shore lines Rescue

**Table 2:** Dependency annotation examples.

Sentence	Grammatical Structure
1. “The two ships collided at 4 am on 27 January 2017.”	S: ships, P: collided
2. “In the morning, the Kamarajar port authority released a press statement that there was no damage to the environment and no casualties or injuries to persons.”	S: authority, P: released, O: statement
	S: damage, P: is
	S: casualty, P: is

**Table 3:** Modifier dependence type.

Modifier Dependency Type	Enhance++ UD Type	Syntactic Relation Example	Phrase
Negation modifier (neg)	neg	neg (casualties, no)	No casualties
Numeric modifier (nummod)	nummod	nummod (tons, 40)	40 tons
Adjectival modifier (amod)	amod	amod (ship, red)	Red ship
Nominal modifier (nmod)	nmod	nmod: to (floats, shore)	Oil floats to the shore
Adv modifier (advmod)	advmod	advmod (rose, high)	Flames rose 100 m high

**Table 4:** Relation triple examples.

Sentence	Relation Triple
1. “The two ships collided at 4 am on 27 January 2017.”	Collided (ships, 2017-01-27T04:00)
	Num (ships, 2)
2. “In the morning, the Kamarajar port authority released a press statement that there was no damage to the environment and no casualties or injuries to persons.”	Released (Kamarajar port authority, press statement)
	Release a press statement (Kamarajar port authority, morning)
	Damage (environment, no)
	Injury (persons, no)

**Table 5:** The 10 selected significant oil spill events since 1967.

Ship name	Year	Location	Spill Size (Tonnes)
ALTANTIC EMPRESS	1979	Off Tobago, West Indies	287,000
ABT SUMMER	1991	700 nautical miles off Angola	260,000
CASTILLO DE BELLVER	1983	Off Saldanha Bay, South Africa	250,000
AMOCO CADIZ	1978	Off Brittany, France	223,000
HAVEN	1991	Genoa, Italy	144,000
ODYSSEY	1988	700 nautical miles off Nova Scotia, Canada	132,000
TORREY CANYON	1967	Scilly Isles, UK	119,000
SEA STAR	1972	Gulf of Oman	115,000
SANCHI	2018	Off Shanghai, China	113,000
IRENES SERENADE	1980	Navarino Bay, Greece	100,000

**Table 6:** Event splitting results.

Ship Name	Total scenario Count	Extracted Scenario Count
ALTANTIC EMPRESS	6	6
ABT SUMMER	2	2
CASTILLO DE BELLVER	2	2
AMOCO CADIZ	1	1
HAVEN	3	2
ODYSSEY	1	1
TORREY CANYON	9	9
SEA STAR	1	1
SANCHI	2	2
IRENES SERENADE	2	2
